# Combining Inulin Multifunctional Polycation and Magnetic Nanoparticles: Redox-Responsive siRNA-Loaded Systems for Magnetofection

**DOI:** 10.3390/polym11050889

**Published:** 2019-05-15

**Authors:** Carla Sardo, Emanuela Fabiola Craparo, Barbara Porsio, Gaetano Giammona, Gennara Cavallaro

**Affiliations:** Dipartimento di Scienze e Tecnologie Biologiche Chimiche e Farmaceutiche (STEBICEF), Università di Palermo, Via Archirafi 32, 90123 Palermo, Italy; csardo@cbm.pan.pl (C.S.); emanuela.craparo@unipa.it (E.F.C.); barbara.porsio@unipa.it (B.P.); gaetano.giammona@unipa.it (G.G.)

**Keywords:** inulin, cystamine, DETA, siRNA, SPIONs

## Abstract

Superparamagnetic Iron Oxide Nanoparticles (SPIONs) are recognized as one of the most promising agents for theranostic applications. Among methods designed for siRNA delivery, magnetofection, that is, nucleic acid cell uptake under the influence of a magnetic field acting on magnetic nucleic acid vectors, is emerging as a unique approach to combining advantages such as strong improvement of the kinetics of the delivery process and the possibility of localizing nucleic acid delivery to an area where the magnetic field is applied. This paper reports on the preparation of siRNA loaded magnetoplexes—named ICD@SS@SPIONs/siRNA—by controlled crosslinking, in the presence of SPIONs, of the polycation INU-C-DETA, synthesized starting from the polysaccharide inulin by grafting diethylenetriamine and cystamine molecules. The obtained ICD@SS@SPIONs/siRNA have suitable chemical-physical characteristics to be employed for iv administration and are also able to release siRNA in a redox-triggered manner thanks to intracellular glutathione (GSH) mediated reduction of disulphide bridges formed during the crosslinking process. Moreover, ICD@SS@SPIONs/siRNA are able to produce magnetic targeting in vitro on breast cancer cells, without appreciable cyto- and hemo-toxic effects, in a wide range of concentrations. Finally, protein binding to nanoparticles revealed that obtained systems are potentially longer circulating and applicable as a smart multifunctional agents for cancer therapy.

## 1. Introduction

Small interfering RNA (siRNA) duplexes are oligonucleotides composed of usually 20–25 base pairs in length that can regulate silencing of the target genes by triggering sequence-specific mRNA degradation [1]. The use of siRNA mediated gene silencing has recently emerged as a promising therapeutic strategy for the treatment of various diseases including cancer, by down-regulating the expression of relevant target genes in a precise and specific manner [2,3]. Despite their high therapeutic potential, siRNAs suffer from degradation by ubiquitous RNase and moreover their high molecular weight, hydrophilicity and negative charge prevent them to adequately penetrate cell membranes [4]. In order to allow an effective intracellular delivery of siRNA, by avoiding degradation, the development of an effective carrier with proper peculiarities is essential. Therefore, a magnetic carrier could be used to carry siRNA inside cells by using magnetofection, that is, by applying a magnetic field to improve nucleic acid cell uptake.

Magnetofection is a recent method designed for nucleic acid based drugs delivery, full of promising advantages such as: (1) potential to increase the response to the administered nucleic acid dose; (2) strong speed in the delivery process; and (3) the possibility of concentrating the administered nucleic acid locally in an area where the magnetic field is applied [5,6]. Moreover, in order to improve the intracellular release of drug, the number of drug formulations that incorporate disulphide bonds is increasing particularly in the next generation macromolecular pharmaceuticals [7,8]. These are designed to exploit differences in the reduction potential at different locations such as cytoplasm that contains a higher concentration of glutathione tripeptide than that in the extracellular fluids or circulation [9]. Furthermore, a particular feature of the tumour tissues is the presence of a highly reducing environment compared with the normal ones, with a glutathione (GSH) concentration at least 4-fold higher than the non-tumour tissues [9], rendering the bioreducible systems a valid and promising approach for tumour-specific drug and gene delivery. The Inulin-Cystamine-Diethylenetriamine (INU-C-DETA) copolymer investigated here was constructed starting from inulin, which is a unique natural polysaccharide, because of its linear backbone that makes it essentially very flexible compared to other polysaccharides [10,11,12]. This is one of the reasons why inulin derivatives could be good candidates for siRNA delivery. In fact, since siRNA molecules have a lower negative density charge in comparison with longer DNA molecules, such as pDNAs [13], siRNA has to be complexed by flexible and high binding polymers [14]. This avoids the need for a high content of positive charges on the vehicle structure that will negatively influence the toxicity profile of the overall system. Grafting of diethylenetriamine (DETA) molecules on inulin backbone leads to a positively charged copolymer that can interact with SPIONs surface and retain siRNA, whereas the introduction of pendent cystamine (C) moieties ensures the stability of the copolymer against reticulation before use; the resultant pendent thiols can be crosslinked by the reduction of disulphide groups in the presence of either siRNA or SPIONs, leading to the formation of tight and stable polymer-coated Super Paramagnetic Iron Oxide Nanoparticles, which are indicated as ICD@SS@SPIONs/siRNA. In this way, the contradictory requirements of efficient non-viral delivery agents of siRNA could be resolved, that is, capability to excellently bind and protect nucleic acids during passage and stay in extracellular fluids and efficient intracellular release of them after redox-triggered disulphide cleavage until culminate to efficient mRNA knockdown. The crosslinked ICD@SS@SPIONs/siGL3 described here show efficient gene silencing thanks to the synergistic contribution of both magnetic targeting and siRNA release by a redox responsive behaviour. These results, associated with a void in vitro cyto- and haemo-toxicity, allow the proposal of these nanoparticles as an innovative strategy for intracellular siRNA delivery.

## 2. Materials and Methods

### 2.1. Materials

Materials, Cell Lines Culture and Reagents are reported in Supplementary Materials.

### 2.2. Synthesis and Characterization of Inulin-Cystamine-Diethylenetriamine Copolymer (INU-C-DETA)

Before use, inulin was dried in an oven at 70 °C for 24 h. Dried inulin (250 mg, 1.5 mmoles of fructose) was dissolved in anhydrous dimethylsulfoxide (a-DMSO, 4 mL) and, after the addition of 4-BNPC (353 mg in 2 mL of a-DMSO), the obtained solution was put on a CEM Discover Microwave Reactor. The irradiation was carried out for 1 h at 25 W and during this time, the temperature of the reaction vessel was kept under 60 °C by cooling with compressed air. Then the reaction mixture was slowly added dropwise to a C solution in a-DMSO, previously treated with 1 equivalent of TEA, in an equal molar quantity to the mmoles of inulin repeating units. The reaction mixture was then kept at room temperature for 2 h, under constant magnetic stirring and then added to a DETA solution (620 µL of DETA in 1 mL of a-DMSO). After other 2 h at room temperature the mixture was precipitated in 200 mL of acetone. The obtained suspension was centrifuged (10,000 rpm, 4 °C) using a Heraeus Megafuge 16R centrifuge (Thermo Fisher Scientific, Waltham, MA, USA) and the residue was washed twice with the same solvents. The obtained solid was carefully dried and then solubilised in distilled water to be purified by gel filtration chromatography using a Sephadex^®^G-25 (GE Healthcare Bio-Sciences, Pittsburgh, PA, USA) as gel filtration medium and double-distilled water as eluent. After recovering, the solution was freeze-dried. 

*^1^H-NMR Characterization*. The pure INU-C-DETA was characterized by ^1^H-NMR analysis using a Bruker Avance II 300 spectrometer (Bruker Corporation, Billerica, MA, USA). ^1^H-NMR (300 MHz, D_2_O, δ ppm): 2.7 (m, 4H_Deta_, –C**H_2_**–NH–C**H_2_**–), 2.8 (m, 2H_Deta_, –C**H_2_**–NH_2;_ m, 2H_Cystamine_, –SS–CH_2_–C**H_2_**–NH_2_), 2.9 (m, 4H_Cystamine_, –O–CO–NH–CH_2_–C**H_2_**–SS–C**H_2_**–CH_2_–NH_2_), 3.2 (m, 2H_Deta_, –O–CO–NH–C**H_2_**–), 3.4 (m, 2H_Cystamine_, –O–CO–NH–C**H_2_**–CH_2_–SS–), 3.5–3.9 (m, 5H_Inu_, –C**H**_2_–OH; –C**H**–CH_2_–OH; –C–C**H_2_**–O–), δ 4.0 (t, 1H_Inu_, –C**H**–OH), δ 4.2 (d, 1H_Inu_, –C**H**–OH).

*SEC analysis*. The weight average molecular weights (*M_w_*) and polydispersity (*M_w_*/*M_n_*) of INU and INU-C-DETA were determined by SEC in aqueous medium, by using two TSK gel column (G4000 PWXL and G3000 PWXL) from Tosoh Bioscience (Tosoh Bioscience Inc., San Francisco, CA, USA), connected to a Waters 2410 refractive-index detector (Waters, Mildford, MA, USA). As eluent a phosphate buffer solution 0.1 M at pH 3.5 was used, at 35 °C and at a flow rate of 0.6 mL/min. To construct the calibration curve, pullulan standards were used (in the range 180–112,000 Da), dispersing them in the mobile phase (2.5 mg/mL) and injected after filtration with 0.45 µm cellulose acetate syringe filters. 

*Primary Amine Quantification*. In order to quantify total inulin functionalization with both DETA and C, the TNBS assay was performed. Briefly, 20 µL of TNBS was added to 25 µL of a 2 mg/mL solution of the sample (INU-C-DETA), solubilised in a tetrahydroborate 1 M solution and diluted to a final volume of 1 mL with bi-distilled water. The absorbance was read at 420 nm and compared with a mono amine PEG (MW: 2000 Da) calibration curve.

*Quantitation of Thiol and Disulphide Groups.* Quantitation of thiol and disulphide groups was performed according to Thannhauser [15,16].

### 2.3. Preparation of ICD@SS@SPIONs, ICD@SS@SPIONs/siGL3(Cy5) and ICD@SPIONs

INU-C-DETA (100 mg, 0.026 mmoles of S–S) was dissolved in 3 mL of a 5 mg/mL TCEP water solution at pH 7.5. This solution was then dialysed against water, using a 500–1000 Da MWCO dialysis tube for 2 h. After this time SPIONs solution (2 mL, 5 mg/mL in water) was added and the dialysis was continued for further 2 h. The obtained dispersion was sonicated for 15 min, treated with hydrogen peroxide in water (1 mL, 0.026 mmol/mL) and sonicated for further 15 min. Finally, dispersion was dialysed for 30 min against bi-distilled water pH 8 and for further 24h against bi-distilled water using 12–14,000 Da MWCO dialysis tube. The solid product was obtained after rapidly freezing the dispersion samples in liquid nitrogen and immediate lyophilisation using the Labconco Freeze Dry System (−48 °C, 80 × 10^−3^ mBar; Labconco, Kansas City, MO, USA). 

siRNA incorporation was obtained with the same procedure, by adding siGL3 aqueous solution (400 µL, 100 µM) or siGL3-Cy5 solution (800 µL, 50 µM) after SPIONs were added. As control, un-crosslinked ICD@SPIONs were formed following the same procedure described above but without the employment of any reducing agent and without treatment with hydrogen peroxide.

### 2.4. Quantitative Evaluation of siRNA Loading into ICD@SS@SPIONs/siGL3 Nanoparticles

An exactly weighted amount of ICD@SS@SPIONs/siGL3 were dispersed in RNase free DPBS at the concentration of 0.2 mg/mL. 50 µL of this sample were then mixed with 50 µL of a solution 0.1 mg/mL DTT and 0.2 mg/mL heparin in DPBS. After 1 h 50 µL of EtBr solution 5 × 10^−3^ mg/mL in DPBS were then added and after further 15 min the fluorescence intensity was measured using a plate reader at an excitation wavelength of 530 ± 25 nm and emission wavelength of 590 ± 10 nm. The amount of loaded siRNA was calculated by a calibration curve of siRNA standards treated the same as samples.

### 2.5. Dynamic Light Scattering (DLS) Measurement and Z-potential Analysis

DLS measurements for dimension and Z-potential studies were performed at 25 °C using a Zetasizer NanoZS instrument (Malvern Instrument, Rome, Italy), fitted with a 532 nm laser at a fixed scattering angle of 173°, using Dispersion Technology Software 7.02 (Malvern Instrument, Rome, Italy). Analyses were carried out on aqueous dispersion of ICD@SPIONs, ICD@SS@SPIONs and ICD@SS@SPIONs/siGL3. The average hydrodynamic diameter (in intensity) and polydispersity index (PDI) were obtained by cumulant analysis of the correlation function. The zeta potential was calculated from the electrophoretic mobility by using the Smoluchowsky relationship and by assuming that K·a >> 1 (where K and a are, respectively, the Debye-Hückel parameter and particle radius).

### 2.6. Scanning Electron Microscopy (SEM)

Freeze-dried ICD@SS@SPIONs samples were visualized by using a scanning electron microscope ESEM XL30 (Philips, Amsterdam, The Netherlands). Powder was dusted on a double sided adhesive tape and then putted on a stainless steel stub, then sputter-coated with gold prior to examination.

### 2.7. FT-IR Analysis

The general compositions of pure solid INU-C-DETA copolymer, ICD@SS@SPIONs and magnetite (Fe_3_O_4_ powder) were analysed via Fourier Transform infrared spectroscopy (FT-IR) in the frequency range of 4000–400 cm^−1^ by using a Brucker Spectrum FT-IR ATR System spectrophotometer (Bruker Corporation, Billerica, MA, USA). Spectra were recorded in reflectance scale with a resolution of 1 cm^−1^ and a number of scans = 100. 

### 2.8. Total Iron Determination

The iron content in ICD@SS@SPIONs and ICD@ SPIONs was measured using a modified ferrozine-based spectrophotometric iron estimation method [17]. Freeze-dried samples (5 mg) were dispersed in 1.4 N hydrochloric acid (0.1 mg/mL) and kept for 2 h at 60 °C in a water bath to allow the mineralization of nanoparticles and the complete dissolution of the Fe_3_O_4_ nanoparticles. After that, the sample solution was diluted 50 times with HCl 0.01 M and then an aliquot of 50 µL was mixed with 50 µL of acetate buffer at pH 4.5 and 50 µL of iron-detection reagent (6.5 mM ferrozine, 6.5 mM neocuproine and 1 M acid ascorbic in acetate buffer at pH 4.5). Samples were incubated for 30 min at room temperature and the absorbance was measured at 570 nm using an AF 2200 plate reader (Eppendorf, Hamburg, Germany). The concentration of Fe_3_O_4_ was calculated by comparing this value with a calibration curve obtained by recording the absorbance of FeCl_3_ standard solutions in HCl 0.01 N (ranging from 3 to 33 µg/mL). 0.01 N HCl with iron-detection reagent was used as blank.

### 2.9. RNase A Protection Assay

To measure the ability of ICD@SS@SPIONs to protect loaded siRNA from degradation due to the nuclease action, the increase in absorbance at 260 nm (hyperchromic effect) was monitored. ICD@SS@SPIONs/siRNA dispersion in ultrapure nuclease free water (120 µL) at a siRNA concentration of 4.16·ng/µL were placed in a UV-VIS 96 well plate (Eppendorf, Hamburg, Germany). Five ng of RNase A (70 U/mg) was then added to 80 µL of water and enzymatic degradation was monitored for 24 h by measuring absorbance at 260 nm at various time intervals on an AF 2200 plate reader (Eppendorf, Hamburg, Germany). Obtained data were reported by plotting the increment % of absorbance at 260 nm versus incubation time (in minutes), where each value represents mean ± standard deviation (n = 9).

### 2.10. DLS with and without GSH

ICD@SS@SPIONs were dispersed in DPBS pH 7.4 or DPBS pH 5 at a concentration of 0.5 mg/mL. To one mL of these solutions 0.2 mL of a GSH solution in DPBS at a concentration of 8.28 × 10^−3^ mg/mL or 18.42 mg/mL, in order to obtain a final GSH concentration of 4.5 µM or 10 mM respectively, were added. At scheduled time intervals within 3h, size was recorded by DLS measurements. 

### 2.11. siRNA Release Study with and without GSH

siRNA release profiles from the crosslinked nanoparticles were quantified by evaluating the amounts of released siRNA at schedule times and by calculating the ratios between these values and the starting amount of loaded siRNA into the ICD@SS@SPIONs/siGL3 as a function of time. The chosen method was the dialysis method: an exactly weighted amount of ICD@SS@SPIONs/siGL3-Cy5, corresponding to 50 µg of siGL3-Cy5 was dispersed into 1 mL of DPBS at pH 7.4 enriched or not with GSH at two different amounts, that is, 4.5 μM that represents the GSH concentration in the extracellular medium, while 10 mM that mimics the intracellular GSH concentration. Samples were transferred into cellulose ester dialysis membranes with a molecular weight cut-off of 1000 KDa (Spectra/Por^®^ Float-A-Lyzer^®^ G2 Dialysis Device. SpectrumLabs now Repligen, Waltham, MA, USA) and were immediately immersed in 50 mL of the same medium. Release studies were conducted in an orbital shaker (37 °C, 100 rpm) and at scheduled time intervals (1 to 26 h) 1 mL of the external medium was withdrawn from the acceptor and replaced with the same volume of clean medium. Samples were freeze dried and dispersed in 180 µL of nuclease free water prior to measurement of the fluorescence intensity by using a RF-5301 PC spectrofluorophotometer (Shimadzu, Kyoto, Japan. Chosen wavelengths: λ_ex_: 649 nm; λ_em_: 673 nm). Values were corrected considering the sample dilution procedure. To calibrate the spectrofluorophotometer and quantify the released siRNA, siGL3-Cy5 standards were prepared in DPBS at a concentration range between 1000–10 nM by serial dilution and were then analysed. Each experiment was performed in triplicate.

### 2.12. Steady State Fluorescence Spectroscopy

Aliquots of Bovine Serum Albumin (BSA) dispersion (50 µL, 660 µg/mL) in 154 mM PBS were added to ICD@SS@SPIONs or ICD@SS@SPIONs/siGL3 dispersion (50 µL in 154 mM PBS) at various concentration in order to obtain nanoparticles concentration in the range 0–100 µg/mL. After 6h at 37 °C under orbital shaking, the emission spectra of the samples were recorded between 300 and 600 nm at a λ_ex_ of 295 nm. The binding data were determined accordingly with Equation (1) which describe the relationship between fluorescence quenching and the binding constant [18,19]:

F0-F/F-FS = Kb·n·[NPs] (1)

where [NPs] indicate ICD@SS@SPIONs or ICD@SS@SPIONs/siGL3 concentration, F0 and F are the relative fluorescence intensities of the solution of BSA alone and BSA solution in the presence of a given amount of ICD@SS@SPIONs or ICD@SS@SPIONs/siGL3 respectively and FS is the relative fluorescence intensity of BSA saturated with nanoparticles. Relative fluorescence intensities were obtained from the area under the fluorescence curve, while FS was extrapolated as the y axis intercept by plotting l/(F0-F) against l/[NPs] (see Supplementary Materials Figure S4); values for FS for ICD@SS@SPIONs and ICD@SS@SPIONs/siGL3 resulted equal to 7.06 × 10^−6^ and 1.22 × 10^−3^ respectively. The number of binding sites (n) and binding constants (Kb) were calculated by plotting log[(F0-F)/(F-FS)] vs log[NPs]. In particular, the Equations (2) and (3) have been obtained by plotting data relative to the experiments in the presence of ICD@SS@SPIONs and ICD@SS@SPIONs/siGL3 respectively:

log[(F0-F)/(F-FS)] = 1.655 log[ICD@SS@SPIONs] − 2.9576(2)

log[(F0-F)/(F-FS)] = 0.8218 log[ICD@SS@SPIONs/siGL3] − 3.6541 (3)

From Equations (2) and (3), the logarithm of the dissociation constant (log K_diss_) has been calculated, considering that log[NPs] = log K_diss_ when log[(F0-F)/(F-FS)] = 0. Kb have been calculated as 1/K_diss_; (n) was obtained from the slope of log[(F0-F)/(F-FS)] vs log[NPs] curves described by Equations (2) and (3).

### 2.13. Size and Zeta Potential as a Function of Albumin Concentration

Aliquots of albumin aqueous dispersion (at a concentration equal to 5.5 mg/mL) were added to aqueous dispersion of ICD@SS@SPIONs and ICD@SS@SPIONs/siGL3 until reaching a concentration of 1 mg/mL. Then, both size and ζ potential values were measured at each albumin concentration increment (0.1 mg/ml) by using a Zetasizer Nano ZSP equipped with an MPT-2 autotitrator (Malvern Instrument, Rome, Italy).

### 2.14. Determination of Relative Albumin Amount Bonding Nanoparticles

In a typical experiment, ICD@SS@SPIONs and ICD@SS@SPIONs/siGL3 were dispersed in 154 mM PBS at a concentration of 5.5 mg/mL. Five hundred microliters of each dispersion was then added to 1 mL of a BSA dispersion in PBS (60 mg/mL) inside RNasi free polypropylene tubes and was incubated for 6 h. Then, samples were centrifuged twice at 14,500 rpm (14,100× *g*) using a MiniSpin^®^ plus (Eppendorf, Hamburg, Germany) to recover BSA bound nanoparticles as pellet. The latter were dispersed in 150 µL of bi-distilled water and 50 µL aliquots were analysed for BSA quantification. For protein content determination 5 µL of SDS solution in water (220 mg/mL) were added to the samples and the final dispersion was heated to 95 °C for 5 min for the BSA to be separated before analysis by BCA kit (Sigma Aldrich, Milan, Italy). Results were expressed as µg BSA per mg of NPs.

### 2.15. In Vitro Gene Knockdown

MCF-7/Luc cells were cultured for 24 h in DMEM containing serum, seeded in 96-well plates at 2 × 10^4^ cells/well before the transfection study. Then, the medium was replaced by serum free OPTI-MEM (200 μL/well) containing ICD@SS@SPIONs/siGL3 nanoparticles at a final siRNA concentration of 200 nM. After incubation at 37 °C for 4 h, the medium was replaced by complete DMEM containing 10% FBS and cells were further incubated for 24 h before lyses and quantification of luciferase expression using the Luciferase Assay System (Promega, Madison, WI, USA), according to the product manual, on a GloMax 20/20 Luminometer (Promega) and measurement of the cellular protein level was made with a BCA kit (Sigma Aldrich, Milan, Italy). The silencing efficiency was expressed as the percentage expression levels of luciferase in control cells that did not were incubated in the presence of siRNA. Identical experiments were performed for naked siRNA at the same concentration. In a parallel experiment, ICD@SS@SPIONs/siGL3 nanoparticles were incubated in the presence of permanent magnet (attraction force of 250 g) under the wells for the entire incubation period. Transfection efficiencies of ICD@SS@SPIONs/siGL3 with and without magnetic field application were related to each other and to naked siRNA. Obtained data were expressed as relative mean values of replicate of 3 ± standard deviation (% of cells with full luciferase expression) normalized by protein content (RLU/mg protein %). 

### 2.16. In Vitro Cell Uptake

1.2 × 10^5^ MCF-7 cells per well were seeded in 24-well plates and incubated for 24 h. Then, the medium was replaced with 0.6 mL of fresh OPTI-MEM I medium containing ICD@SS@SPIONs/siGL3-Cy5 nanoparticles at a final siRNA concentration of 200 nM. After 4 or 24 h, cells were washed three times with DPBS and lysed in 100 μL lysis buffer (1% Triton X-100, 2% SDS in DPBS) at −20 °C. Seventy-five µL of lysates were transferred to quartz cuvette for fluorescence intensity measurements (λ_EX_: 647 nm; λ_EM_: 673 nm) on a spectrofluorophotometer (RF-5301 PC Shimadzu, Kyoto, Japan). 25 μL of lysates were used in order to determine the protein content trough the bicinchoninic acid kit for protein determination (Sigma Aldrich, Milan, Italy), according to the manufacturer’s protocol. For determination of the mean fluorescence intensity, fluorescent signals were corrected for the amount of protein in the samples. The same experiment was carried out by applying a permanent magnet (attraction force of 250 g) under the wells for the entire incubation period. Results were compared with each other and with cells treated with 200 nM siRNA in OPTI-MEM I medium. The experiment was carried out in triplicate.

### 2.17. Statistical Analysis

In order to compare different groups, a T-Test was applied. Reported data were assumed statistically significant with a value of *p* < 0.05. All values were calculated as the average of three experiments ± standard deviation. 

## 3. Results and Discussion

### 3.1. Synthesis and Characterization of INU-C-DETA Copolymer

The INU-C-DETA copolymer here investigated, was synthesized starting from inulin [20], by grafting two different molecules: diethylenetriamine (DETA) and cystamine (C). 

The functionalization of the polymer backbone with DETA molecules lead to obtaining a graft copolymer bearing 1,2-diaminoethane moieties in the side chains that show a pKa between 8 and 10 [21], which confers the characteristic of polycation to the final structure when at physiological pH. Amines can also interact with SPIONs surface, giving the possibility to form a hydrophilic coating that increases colloidal stability and the in vivo circulation by reducing opsonization and uptake by the mononuclear phagocyte system [22]. In addition, 1,2-diaminoethane residues show a peculiar two-step protonation behaviour that could allow a membrane destabilization of the acidic pH of late endosome or lysosome thank to its conformation change, thus enabling escape to the cytoplasm [23].

Pendent cystamine moieties contain a disulphide bond that is prone to being converted into thiol groups after proper reduction, while ensuring in the disulphide form the stability of the copolymer before use. Crosslinking of resultant pendent thiols to form intra- and inter-molecular disulphide bonds in the presence of Super Paramagnetic Iron Oxide Nanoparticles (SPIONs) lead to the formation of tight and stable polymer-coated ICD@SS@SPIONs. Cytosol, as well as other intracellular compartments (mitochondria and cell nucleus) contain a high concentration of glutathione (GSH) tripeptide (about 2–10 mM), which is 100 to 1000 times higher than the concentration found in the extracellular fluids and circulation (about 2–20 μM); the introduction of redox responsive crosslinking could enable the binding and protection of nucleic acids in extracellular fluids and the efficient release of nucleic acids inside the cells, by a prompt release of siRNA in the cytosol upon redox-triggered disulphide cleavage.

Finally, the presence of thiols in a biomaterial provide the possibility of easily introducing a small amount of different moieties to tailor the structure for different applications (specific targeting ligand, bioactive molecules, hydrofobizing agent, fluorescent probes, etc.) by convenient reactions such as thiol-ene and thiol-yne reactions, which have recently been significantly upgraded due to the recognition of their “click” characteristics [24,25,26].

**Scheme 1:**
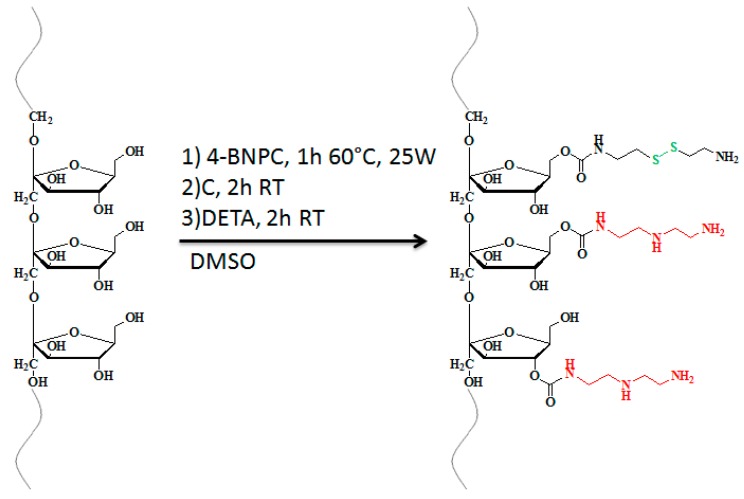
Schematic representation of the synthesis of INU-C-DETA copolymer.

INU-C-DETA was obtained according with a synthetic protocol already used to obtain other INU based polycation [17,27,28], involving activation of free inulin hydroxyl groups with 4-BNPC by Enhanced Microwave Synthesis (EMS), a very high yielding, easy and scalable approach for post polymerization functionalization [29]. INU-C-DETA structure and synthesis conditions are graphically represented in Scheme 1.

**Figure 1:**
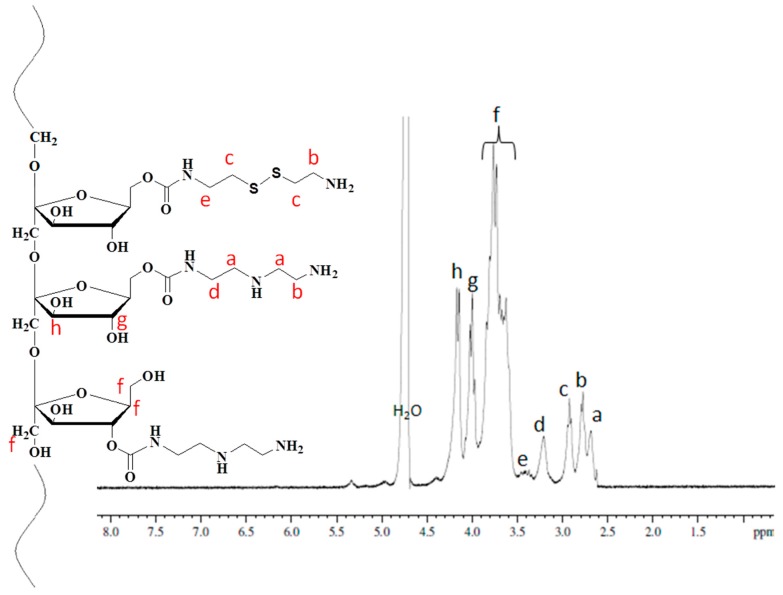
Representative ^1^H-NMR spectrum of INU-C-DETA in D_2_O.

^1^H NMR analysis of INU-C-DETA derivative (Figure 1) showed new peaks in comparison with native inulin, at δ comprised between 2.5 and 3.5 ppm, that confirms the introduction of DETA and C functionalities in this derivative. Although it is not possible to quantitatively discriminate between C and DETA contributions in ^1^H NMR spectrum, a total degree of functionalization in C and DETA portions of fructose repeating units was obtained by comparing the integrals of all signals between 2.5 and 3.5 ppm attributed to the sum of protons of linked C and DETA portions with that attributed to inulin protons between 3.5 and 4.5 ppm; this value was found to be equal to 25 mol% respect to fructose inulin repeating units. In order to properly characterize the obtained copolymer, specially analytical methods were employed for –S–S– and –NH_2_ groups determination.

The quantification of total primary amines, of both DETA and C pendants, has been performed by TNBS assay and the value obtained, equal to 25 mol%, confirmed the ^1^H-NMR determination of the overall fructose repeating unit’s functionalization. The amount of C moieties bounded to inulin was also determined by disulphide groups’ quantification with the Thannhauser assay, which revealed a 5 ± 3 mol% inulin functionalization with C. By subtracting this value to the total DD_mol_%, a DETA molar functionalization of 20 ± 2 mol%, with respect to fructose inulin repeating units, was obtained. The average weighted molecular weight of INU-C-DETA, determined by SEC analysis in acidic aqueous media, was 6217 Da, with a polydispersity (*M_w_*/*M_n_*) of 1.83. By comparing calculated *M_w_* (6173 Da), considering performed chemical functionalizations, with that found experimentally, it is possible to reasonably conclude that under the employed conditions, no cross-linking between inulin chains occurred during the functionalization reaction.

Reaction conditions to obtain INU-C-DETA and its molecular characteristics are summarized in Table 1.

### 3.2. Preparation and Characterization of INU-C-DETA Crosslinked Superparamagnetic Iron Oxide Nanoparticles (ICD@SS@SPIONs)

In order to obtain INU-C-DETA crosslinked SPIONs (ICD@SS@SPIONs), a procedure at three subsequent steps was followed. In fact, after a first thiol deprotection, by disulphide reduction with the water-soluble reducing agent phosphine TCEP and purification treatment, the formulation process to obtain ICD@SS@SPIONs included two subsequent steps involving the charge interaction of a positive copolymer with a negative SPIONs surface (ζ-potential in water −29.2 mV) [17,30], followed by oxidative crosslinking catalysed by H_2_O_2_ in a basic environment. This lead to multi iron-cored positive nanoparticles (~9 mV ζ-potential) with an average hydrodynamic diameter (Z-average) of 170 nm and PDI value of 0.093, indicating a very homogenous size distribution.

**Figure 2:**
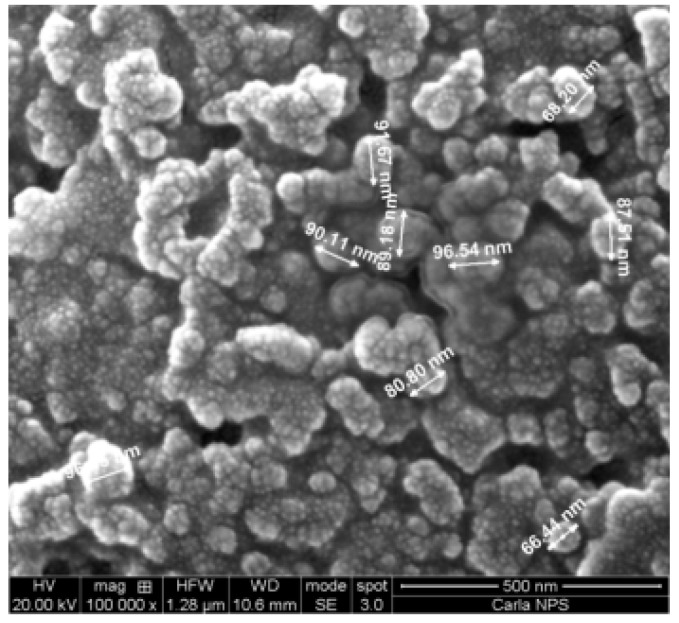
Representative scanning electron microscopy (SEM) image of ICD@SS@SPIONs.

Inspection of the solid formulation obtained by freeze-drying by SEM (Figure 2) gave images highlighting spheroid nanoparticles with an average diameter of 73.93 ± 15.87 nm, which is smaller than the DLS Z-average in aqueous medium; this phenomenon, otherwise observed [17,30], is supposed to be caused by the shrinkage of the coating copolymer upon drying. Moreover, these differences could be justified by the kind of employed technique, being one a direct method of visualization by a microscope (SEM) and the other one an indirect method that comprehend the hydration shell of particles in aqueous dispersion. A formulation obtained by the simple incubation of INU-C-DETA copolymer and SPIONs and therefore, with protected thiols and without the oxidative crosslinking step (uncrosslinked ICD@SPIONs), had a Z-average of 182 nm, a higher polydispersity (PDI 0.174) and a higher positive charged surface (ζ-potential 14 mV) because the primary amines of unreduced cystamine moieties (–S–CH_2_–CH_2_–NH_2_) are still present in the copolymer structure.

**Figure 3:**
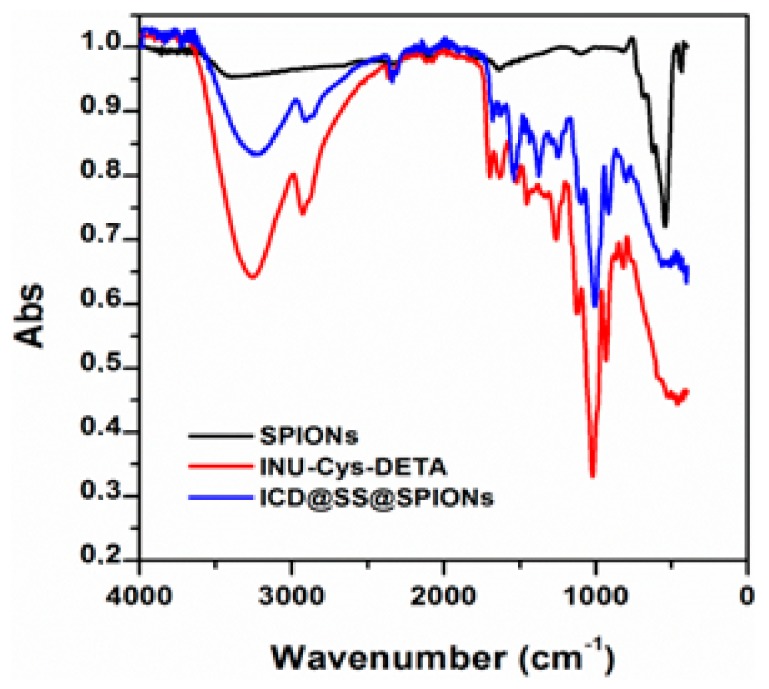
Fourier transform infrared (FT-IR) spectra of SPIONs, INU-C-DETA and ICD@SS@SPIONs.

The existence of the polymeric coating and the chemical composition of the ICD@SS@SPIONs was firstly confirmed by FT-IR. In Figure 3 the FT-IR spectrum of ICD@SS@SPIONs is reported in comparison with that of SPIONs (Iron(II,III) oxide) and INU-C-DETA copolymer alone. The FT-IR spectrum of Iron(II,III) oxide showed the characteristic broad band of Fe–O stretching at 540 cm^−1^, whereas the INU-C-DETA copolymer displays characteristic C–O–C stretching at 1025 cm^−1^, C=O stretching bands at 1630 and 1700 cm^−1^, N–H bending and C–N stretching at 1520 cm^−1^ and a broad band at 3300 cm^−1^ of hydroxyl and amine groups. The spectrum of ICD@SS@SPIONs clearly shows the vibrating bands of the polymer consistent with the existence of a polymer coating on magnetite core. 

Quantitative iron determination by ferrozine colorimetric assay was also in agreement with the conclusion that the sample is effectively constituted by copolymer and magnetite, the latter in a percentage of 4.4% ± 0.5% on a weight basis. A greater percentage of magnetite was found in the uncrosslinked ICD@SPIONs, showing that the used crosslinked coating method allows the formation of nanoparticles with a polymer matrix among magnetite particles. A further confirmation of S–S crosslinked bridges was obtained by Thannhauser’s assay. In particular, the amounts of the S–S and SH groups of ICD@SS@SPIONs were determined and compared with those obtained by uncrosslinked ICD@SPIONs. 

Although half of the thiol groups initially involved in disulphide bond of the cystamine were removed after reduction with TCEP, twice the mmol (SH + SS)/mg% have been found in ICD@SS@SPIONs with respect to the uncrosslinked mixture, as stated through the quantification of thiols and disulphide. Composition information of ICD@SS@SPIONs and ICD@SPIONs are summarized in Table 2.

The superparamagnetic behaviour of prepared ICD@SS@SPIONs was evidenced macroscopically (Figure S1) by the attractive effect of an external magnet on the nanoparticles water dispersion: Figure S1a clearly displays ICD@SS@SPIONs that, after the application of an external magnet for few hours, were easily recovered and accumulate near the magnet. After the removal of the magnetic stimulus, a homogeneous dispersion was re-obtained (Figure S1b), thus indicating the superparamagnetic behaviour of the nanoparticles and the good physical stability of the polymer coating.

### 3.3. Preparation and Characterization of ICD@SS@SPIONs/siRNA Systems

**Figure 4:**
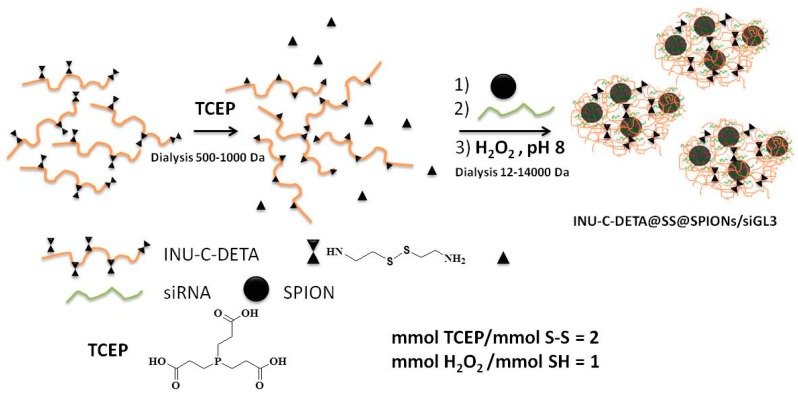
Schematic representation of ICD@SS@SPIONs/siRNA preparation.

The same preparation procedure described above for ICD@SS@SPIONs was followed in order to load siRNA. A schematic representation of ICD@SS@SPIONs/siRNA preparation is reported in Figure 4. In particular a siRNA solution was added after SPIONs addition to the deprotected polymer dispersion into the dialysis tube. During the subsequent dialysis step the system was purified of unloaded siRNA.

By using an INU-C-DETA:siRNA weight ratio of 40:1, an encapsulation efficiency of 50.97% ± 10.10%—which corresponds to a siRNA loading of 1.20 ± 0.33 wt%—was obtained. After siRNA incorporation, ICD@SS@SPIONs/siRNA nanoparticles showed a hydrodynamic diameter of 458 nm (PDI 0.241) and an inverted surface charge (ζ-potential ~ −6 mV), suggestive of siRNA incorporation and neutralization of excess of positive charged groups, was exposed on the nanoparticles’ surface.

The importance of the incorporation process, that is, crosslinking after siRNA interaction, on the siRNA loading efficiency has been proved by conducting complexation ability studies of siRNA by INU-C-DETA or ICD@SPIONs or ICD@SS@SPIONs by simple mixing of sample dispersion with siRNA solutions at different weight ratios between the two components ranging from 5 to 40. Such studies, the results of which are shown in Figure S2, highlighted that by simple mixing conditions no siRNA can be entrapped within both crosslinked and uncrosslinked nanoparticles and that the INU-C-DETA copolymer alone was unable to form itself stable polyplexes. Similar behaviour was observed for other inulin derivatives such as Inulin-Ethylenediamine copolymer (INU-EDA) that, when alone, was not able to form stable polyplexes, whereas when the polymer was used as SPIONs coating a good siRNA complexation was realised [17,20]. The reason for this behaviour could reside in the differences between various interaction processes that are responsible for nanostructure formation, such as polyelectrolyte interaction and the collapse of macromolecules in nanostructured complexes (polyplex formation) instead of the layering of opposite charge macromolecules on a nanoparticle’s surface (adsorption driven by electrostatic interaction). In the present work a physical entrapment driven by crosslinking after adsorption of siRNA is considered as the most probable mechanism leading to siRNA loading into ICD@SS@SPIONs.

### 3.4. Lyophilisation and Ionic Strength Effect on Colloidal Stability

In principle, the ICD coating around iron oxide cores should act not only as a reservoir for loading siRNA molecules but also to confer to the resulting nanoparticles greater bio- and hemocompatibility, colloidal stability in the physiological medium and in the presence of proteins, and it should also prevent the rapid clearance of the system by the reticulo-endothelial system (RES), following intravenous administration; if not, the theranostic application of these systems remains confined to targeting the liver or the organs of the lymphatic system such as the thymus and the spleen. Moreover, colloidal stability of these systems after lyophilization is one of the basic properties to evaluate their real applicability in further in vitro and in vivo studies. For these reasons, size and ζ potential of ICD@SS@SPIONs have been measured after freeze drying process and re-dispersion in pure water or PBS 154 mM buffer at pH 7.4. DLS data are summarized in Table 3. 

After the freeze drying process and water re-dispersion, the ICD@SS@SPIONs maintained the same size and surface charge (Z-average 169 nm; PDI 0.203; ζ-potential ~9 mV), whereas ICD@SPIONs showed higher hydrodynamic diameter and less homogeneous size distribution, in addition to an increased surface charge (Z-average 257 nm; PDI 0.293; ζ-potential 28 mV), indicating a different rearrangement of positive amine moieties on the surface and a poor stability as compared with ICD@SS@SPIONs, where intra- and intermolecular disulphide linkages lead to a tighter and stable nanostructure. 

Also, in the case of ICD@SS@SPIONs/siRNA size, polydispersity and ζ potential were measured after freeze drying—a Z-average value of 466 nm (PDI 0.200) and a ζ-potential of −6 mV were obtained in water. The comparison of these values with that recorded before freeze drying confirmed the physicochemical stability of these obtained nanodevices during lyophilization process. 

In deionised water, that is, in the absence of the preferential adsorption of soluble ions in solution, colloidal stability is dominated by steric stabilization effects and surface ionization, which controls particle surface charge and in these conditions depends on the ionization degree of functional groups at the specific pH. The presence of salts (ionic strength higher than 0.1 M), cause a compression of electrical double layer and, although the particle surface charge may be unchanged when ions do not interact with the particle surface, the zeta potential decreases with increasing ionic strength, decreasing the energy barrier to prevent aggregation. In these conditions, if steric factors are not enough to dominate repulsion, an ionic strength increase will correspond with aggregation phenomena with an increase in particle size and polydispersity index. This is what probably happens in the case of uncrosslinked ICD@SPIONs in PBS 154 mM; having a lower polymer density, the increase in ionic strength causes colloidal instability, as demonstrated by an increase of the Z-average up to 612 nm with a PDI of 0.555 and a ζ-potential of ~0 mV. On the contrary, ICD@SS@SPIONs in PBS 154 mM maintain the same size and narrow dimensional distribution (Z-average 168 nm, PDI 0.151), even with a lower charge-inverted ζ-potential equal averagely to −2.7 mV. Moreover, while after freeze-drying ICD@SS@SPIONs were able to form a colloidal dispersion in high ionic strength culture medium such as DMEM supplemented with 10% FBS, ICD@SPIONs were not dispersible under the same conditions (Figure S3). Upon siRNA loading a slight increase in average size and polydispersity was obtained (Z-average 600 nm, PDI 0.243), without appreciable variation of ζ-potential (−5.6 mV) confirming the overall stability of this siRNA loaded formulation.

### 3.5. Redox Responsiveness of ICD@SS@SPIONs and siRNA Release

To observe destabilization consequent to reduction-triggered breakage of the crosslinked polymer network, the ICD@SS@SPIONs without loaded siRNA were incubated with different concentration of GSH in PBS buffer at pH 7.4 and pH 5.1 at 37 °C and the size changes of these systems was examined at different time intervals by DLS. Conditions were chosen in order to simulate different physiological conditions and moreover in order to take in account the pH sensitive nature of SS/SH equilibrium. In fact, GSH is an intracellular reducing agent that is widely present in the cytosol at a concentration of around 10 mM and less present in the extracellular fluids, with a concentration of around 4.5 µM.

**Figure 5:**
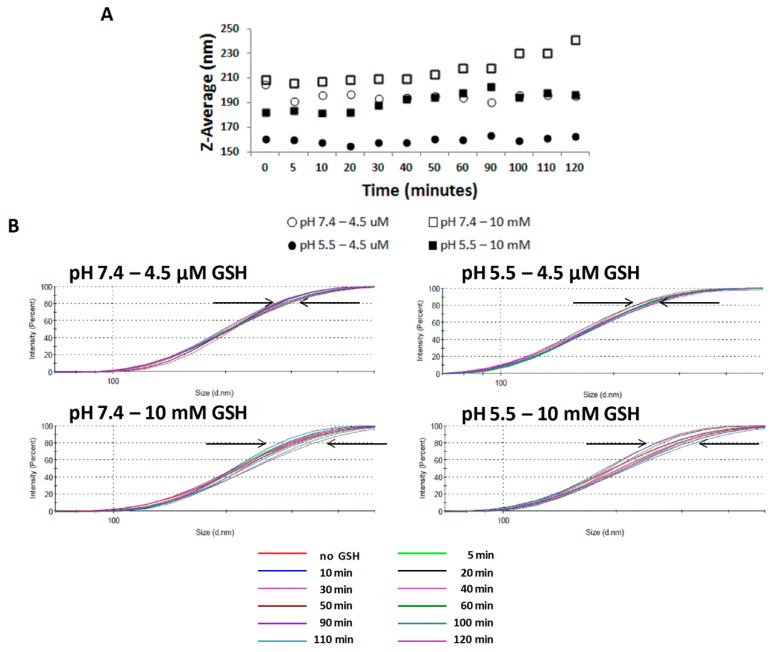
Particle size of ICD@SS@SPIONs upon treatment with GSH 4.5 µM (extracellular environment) and 10 mM (intracellular environment) at two different pH (7.4 and 5.5). (**A**) Z-average distribution over time. (**B**) Cumulative undersize distribution over time.

As can be seen in Figure 5A, average size of ICD@SS@SPIONs did not profoundly changed after 120 min incubation at all considered conditions and stability of the colloidal dispersion remain substantially unaltered. 

Looking at the cumulative undersize distribution, reported in Figure 5B, it is possible to observe that passing from 4.5 µM to 10 mM GSH particle population seemed enriched over time with particles with a larger hydrodynamic diameter. This indicate that, under the experimental conditions used, the cleavage of the disulphide bond occurred leading to the swelling of the coating of ICD@SS@SPIONs. During experiment no smaller peaks indicating the presence of detached copolymer were detected and no further increase in size occurred after 4 h incubation indicating that no disruption of the overall structure of nanoparticles nor aggregation phenomena occurred in these conditions. 

**Figure 6:**
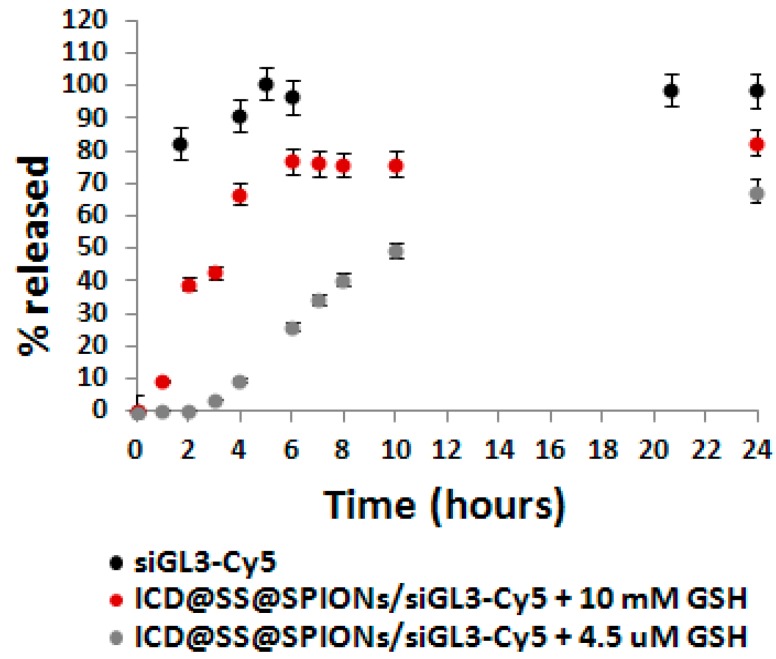
siRNA-Cy5 release from ICD@SS@SPIONs/siRNA-Cy5 in DPBS enriched with 4.5 µM or 10 mM GSH.

The siRNA release profile from ICD@SS@SPIONs/siRNA was also followed by incubating systems containing fluorescent siRNA in DPBS at pH 7.4 at the two GSH concentrations mimicking intracellular (10 mM GSH) and extracellular (4.5 µM GSH) reducing environment. As shown in Figure 6, while in the presence of 4.5 µM GSH release kinetic was very low with only 26% of siRNA released, after 6h in the presence of 10 mM GSH 80% of siRNA was found in the acceptor compartment. This result confirms that ICD@SS@SPIONs/siRNA was able to release siRNA under reducing stimulus thus providing a potential strategy for specific intracellular siRNA delivery.

### 3.6. In Vitro Biological Evaluation: Interaction with Blood Components and Cellular Studies

Although various kinds of cationic polymers can form nanosized polyelectrolyte complexes with negatively charged siRNAs by ionic interactions, their fate when in contact with the biological environment remain a big hurdle. After i.v. administration, nanocomplexes immediately meet red blood cells (RBCs), serum proteins and nuclease enzymes. Interaction with each of these blood components could lead to modification in the colloidal behaviour of the system, degradation of the cargo and side effects, such as haemolysis. To delineate this phenomena and also cytocompatibility and transfection efficiency for our system, we conducted a series of in vitro studies for the evaluation of the interaction with biological components. The results of these studies are presented and discussed in the following sections.

#### 3.6.1. Protein Binding

**Figure 7:**
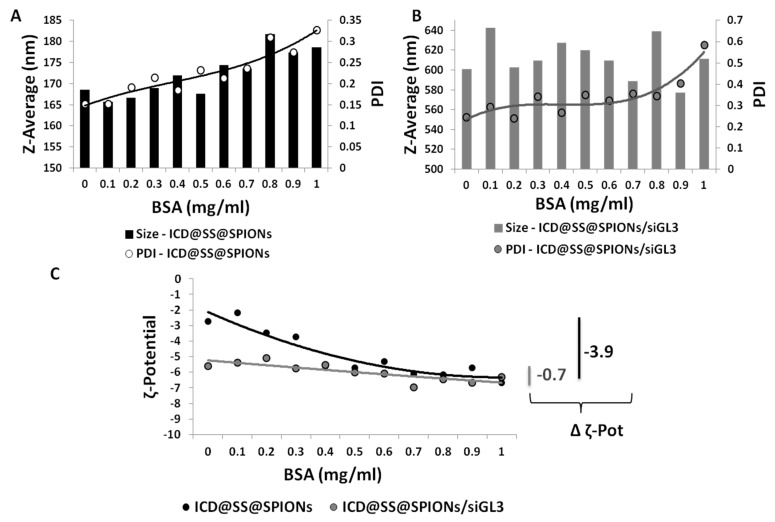
Interaction of bovine serum albumin (BSA) with ICD@SS@SPIONs and ICD@SS@SPIONs/siRNA complex nanoaggregates determined by size (**A**,**B**) and ζ potential (**C**) measurements by DLS.

The absorption of blood serum proteins on nanoparticles can modify their size, aggregation state and interfacial composition and thus offer a distinct “biological identity” able to interfere with clearance, biodistribution and uptake mechanism [31]. The availability of more data on nanomaterials/protein interaction could be useful to compare different systems in vitro by each other or, for example, to build up a classification in which particles can be distinguished in low and high protein binding systems. This, together with the evaluation of other critical parameters such as size, composition, core properties, shell/surface modifications, could help in the rational design and development of nanosystems targeted for an even more specific biomedical application. NPs colloidal stability in a complex media is significantly affected by the adsorption of macromolecules such as proteins [32,33,34]. Therefore, the stability of ICD@SS@SPIONs and ICD@SS@SPIONs/siRNA was investigated in PBS by measuring particle size and zeta potential during titration with BSA, in a concentration range of 0.1–1 mg/mL (Figure 7).

While the average particle size of ICD@SS@SPIONs increased of averagely 10 nm after 1 mg/mL BSA concentration was reached with a slight increase in PDI (Figure 7A), which does not exceed 0.350, in the case of ICD@SS@SPIONs/siRNA marked oscillation of size and a strong increase in polydispersity occurred, up to a PDI of about 0.600 (Figure 7B). 

ζ-potential of both ICD@SS@SPIONs and ICD@SS@SPIONs/siRNA decreased upon BSA addition. This is highly acceptable because BSA is negatively charged at pH 7.4. ζ-potential becomes much more negative for ICD@SS@SPIONs, with a variation of 3.9 points, whereas for ICD@SS@SPIONs/siRNA a variation of only 0.7 points ζ-potential was registered (Figure 7C). 

Tryptophan (Trp), tyrosine (Tyr) and phenylalanine (Phe) are amino acids with fluorophore properties, whose fluorescence is susceptible to quenching upon variation in the microenvironment of the residues; the contribution of Phe to the intrinsic fluorescence of protein is insignificant due to its low absorptivity together with a very low quantum yield. Although Tyr shows a quantum yield similar to Trp, the indole group of Trp is the dominant source in proteins of UV absorbance at 295 nm and emission at ~350 nm. Furthermore, in native proteins, Tyr emission is often quenched, probably due to its interaction with the peptide chain or via energy transfer to Trp [35]. Bovine Serum Albumin (BSA) has two Trp residues (Trp213 and Trp134), which are localised in the hydrophobic region and hydrophilic region respectively [36]. If, after incubation of BSA with ICD@SS@SPIONs or ICD@SS@SPIONs/siRNA, BSA absorption on their surface occurs in the region near to the Trp residues, perturbations in the fluorescence intensity and maximum emission wavelength occur as result of quenching interactions and structural modification, respectively. Moreover, the binding constant (Kb) and the number of binding sites (n) can be determined. 

**Figure 8:**
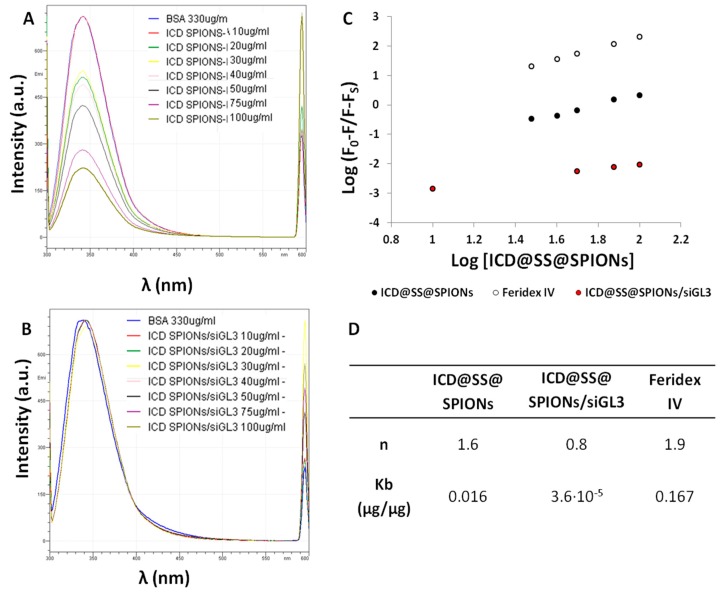
BSA binding on ICD@SS@SPIONs and ICD@SS@SPIONs/siRNA. Representative fluorescence spectral profile (λ_ex_ 295 nm) of BSA dispersion with increasing concentrations of (**A**) ICD@SS@SPIONs and (**B**) ICD@SS@SPIONs/siRNA (0–100 µg/mL). (**C**) Graph based on fluorescence data, where F0, F and FS are the relative fluorescence emission intensity of BSA alone, in the presence of nanoparticles and infinity saturated nanoparticles, respectively. (**D**) Number of binding sites (n) and binding constant (Kb) calculated for ICD@SS@SPIONs and ICD@SS@SPIONs/siRNA compared with n and Kb of Feridex IV, reported by Yallapu et al. [18].

On selective excitation of the Trp residues at 295 nm, for ICD@SS@SPIONs, a marked decrease in the fluorescence emission intensity at 342 nm was observed, which indicates quenching of nanoparticles on BSA (Figure 8A). Although it is reported that higher is nanoparticle size higher will be the extent of protein interaction [31], in the case of ICD@SS@SPIONs/siRNA very little quenching effect has been recorded, corresponding with a low binding (Figure 8B). From the plots of LogF0-F/F0-Fs versus log[NPs] (slope and intercept) the number of binding sites (n) and binding constants (Kb) were calculated (Figure 8C,D).

From this study it is clear that the incorporation of siRNA and the consequent variation in surface charge from positive to slightly negative, limit the BSA interaction, probably because of the negative nature of the protein in PBS at pH 7.4. The difference in the binding constant is substantially high between ICD@SS@SPIONs (Kb = 1.6 × 10^−2^ µg/µg) and the ICD@SS@SPIONs/siRNA loaded counterpart (Kb = 3.6 × 10^−5^ µg/µg), differing by 3 orders of magnitude.

No observation of any red shift of λ_max_ from 342 nm indicates that no substantial structural modification of BSA occurred.

Feridex IV is a commercial formulation, Food and Drug Administration (FDA) approved and composed of super-paramagnetic iron oxide associated with dextran, employed as a magnetic resonance imaging contrast agent for intravenous administration. Feridex IV is taken up by the cells of the reticular-endothelial system (RES), its effect showing as a reduction in signal intensity in non-tumour tissues. This results in image darkening due to signal loss on mid T1/T2 or strongly T2-weighted images. On the other hand, tissues where RES function is decreased (e.g., metastases, primary liver cancer, cysts, benign tumours, adenomas and hyperplasia) retain their native signal intensity, so increasing the contrast between normal and altered tissues.

Yallapu et al. [18] compared Feridex IV with systems composed of SPION core coated by oleic acid functionalised PEG (*M_w_* ranging from 2104 to 8525 Da). These systems showed a lower BSA binding constant (ranging between 5.6 × 10^−2^ and 6.6 × 10^−2^ µg/µg measured in PBS 154 mM) than Feridex IV (0.167 µg/µg) and a long circulation time in vivo with an enhancement of the T(2) MRI contrast, highlighting the possibility of broadening the use of SPIONs for the detection of tumour lesions independent of RES uptake. ICD@SS@SPIONs and ICD@SS@SPIONs/siRNA showed a lower BSA binding than the pegylated systems studied by Yallapu et al., also representing a possible alternative for targeting organs and tissues other than the liver or the organs of the lymphatic system. The lower binding of BSA on ICD@SS@SPIONs/siRNA than on ICD@SS@SPIONs is also confirmed by BSA quantification after protein interaction and purification by centrifugation. Results, expressed as µg BSA per milligram of NPs, showed about two fold increase in protein binding between ICD@SS@SPIONs/siRNA (7 µg BSA/mg NPs) and ICD@SS@SPIONs (13 µg BSA/mg NPs). This is in accordance with the number of binding sites being equal to 0.8 and 1.6 for ICD@SS@SPIONs/siRNA and ICD@SS@SPIONs, respectively.

Despite lower binding properties, siRNA loaded systems have a higher hydrodynamic diameter, which is also a key factor for RES sequestration; for this reason, in vivo studies will be necessary to confirm the stability and long circulation capability of ICD@SS@SPIONs/siRNA. Moreover, the nanoparticles-protein interaction will determine how systems can be best utilized in therapeutics: in fact, different adsorption patterns dictate the target, based on the enrichment on the surface with a specific factor (such as transferrin, apolipoprotein E-1, albumin, etc.) able to give new biological identity to the system and target it in a specific manner [19,31].

The size results, apparently in contrast with binding measurements by fluorescence spectroscopy, demonstrate how BSA, as other serum proteins, could either stabilize NPs through adsorption onto particle surface or destabilize even without a strong binding. It was found that full protein coverage of surfaces can provide additional stabilization, while under certain circumstances, the presence of only a few patches of biomacromolecules is most likely to induce destabilization [32]. Thus, it is possible that, by interacting with ICD@SS@SPIONs, BSA forms a strongly bounded layer (usually indicated as hard corona) that fully covers the surface of NPs, which avoids marked aggregation phenomena, providing steric stabilization in PBS solution. Conversely, in the case of ICD@SS@SPIONs/siRNA, two simultaneous phenomena are possible contributors to the increase in polydispersity and size fluctuations: firstly, BSA exhibited poor and inhomogeneous binding, not enough to form a hard corona (as demonstrated by low Kb and n) but enough to lead PDI increase and size fluctuations due to the fact that instantaneous adsorption/dis-adsorption on NPs have to be considered; secondly, BSA, as a polyanion, could lead to competitive exchange with a loosely bounded fraction of loaded siRNA molecules [32], thus causing a certain degree of destabilization.

#### 3.6.2. Interaction with Red Blood Cells

Toxicity profile on human red blood cells was evaluated by microscopy visualization and haemolysis assay (Figure S5). These data indicate that ICD@SS@SPIONs are haemocompatible and the behaviour was consistent with that reported for other magnetic nanoparticle formulations [19,37].

No sign of toxicity was observed together with negligible haemolysis in the entire tested concentration range. Microscopic images (Figure S5A) depict that no sign of toxicity was observed in the case of the ICD@SS@SPIONs, since RBCs maintained a healthy smooth oval biconcave disc shape structure like negative control (DPBS). Furthermore, this haemocompatible behaviour was confirmed by haemolysis assay (Figure S5B); in fact, negligible haemolysis was observed in the entire tested concentration range. Similarly, after incubation with INU-C-DETA alone, no significant haemolysis was observed (Figure S5D), although at 1000 µg/mL most of RBC showed a lightly echinocyte shape (Figure S5C), that is symptom of lipid membrane alteration and cytoskeleton protein reorganization [38].

#### 3.6.3. siRNA Protection against Nuclease Degradation

**Figure 9:**
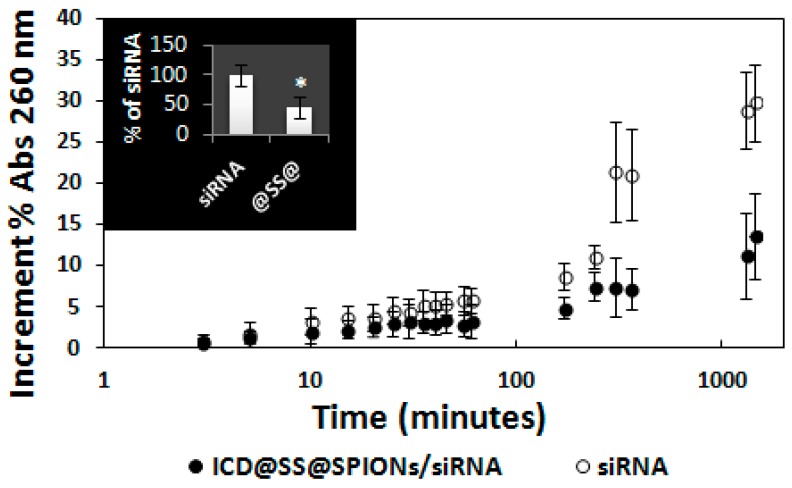
Increments (%) in absorbance at 260 nm of ICD@SS@SPIONs/siRNA dispersion as a function of time. Necked siRNA is reported as positive control. Insert in figure represents the percentage of degraded siRNA after 24 h respect to free siRNA. *p* ≤ 0.005. (Note that after 2 h of dialysis—1000 Da MWCO—almost all the siRNA was able to reach the acceptor compartment).

One of the major barriers to siRNA bioactivity in vivo is the siRNA degradation and inactivation by nuclease enzymes. Here, siRNA protection by ICD@SS@SPIONs has been evaluated by the hyperchromic effect at 260 nm during exposure to RNase (Figure 9).

Results show how after 24 h, free siRNA exposed to the RNase showed a 29.83% increase in Abs_260_, that correspond to a full siRNA degradation. When the experiment was repeated with the same amount of siRNA but enclosed in ICD@SS@SPIONs, after 24 h an hyperchromic effect of only 13.64% was found. These data demonstrate that the incorporation of siRNA inside ICD@SS@SPIONs effectively protected it of some extent against degradation. In particular, the percentage of degraded siRNA after 24 h respect to free siRNA resulted on average of 45%; this fraction of degraded siRNA could be presumably that more exposed on the surface and faster released from ICD@SS@SPIONs.

#### 3.6.4. Cytocompatibility

Cytocompatibility of ICD@SS@SPIONs and INU-C-DETA copolymer was evaluated by the metabolic MTS assay on two different cell lines, in the human bronchial epithelial (16HBE) and in the human breast cancer (MCF-7) cells, after incubation for 48 h. Results, in term of cell viability (%), as a function of samples concentration, are shown in Figure S6a,b. Neither copolymer nor coated SPIONs appeared to produce a cytotoxic effect on both the considered cell lines in the entire range of concentrations tested, since viability results were over 80%.

### 3.7. Transfection Efficiency and Cellular Uptake

**Figure 10:**
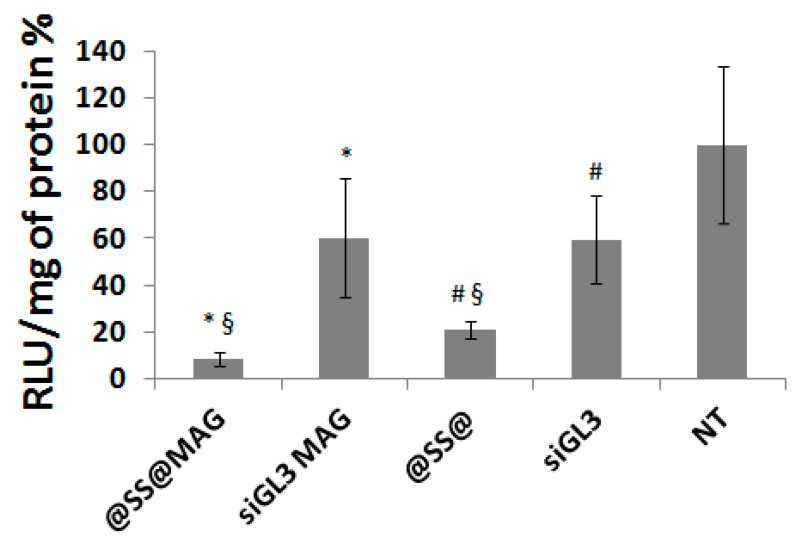
Transfection on MCF-7 cells (4 h) of ICD@SS@SPIONs/siRNA with (@SS@MAG) and without (@SS@) the application of an external magnet. Results were compared with cells treated with free siRNA in the presence (siGL3MAG) or not (siGL3) of the external magnet. A statistically significant difference was found between groups headed by the same symbol (*; #; §) with *p* ≤ 0.05.

To evaluate the transfection efficiency of siRNA encapsulated in ICD@SS@SPIONs, in vitro experiments were conducted on MCF-7/Luc cells under magnetofective conditions. Transfection results are shown in Figure 10. ICD@SS@SPIONs/siGL3 and naked siRNA, used as negative control at the same concentration used for the systems, were incubated with cells after placing magnets under the well’s bottom. The same experiment was conducted without magnet at the bottom of the wells.

This study demonstrates that once inside cells ICD@SS@SPIONs/siGL3 were able to down regulate luciferase expression up to about 80% without magnetic field and up to 90% in the presence of the magnet.

**Figure 11:**
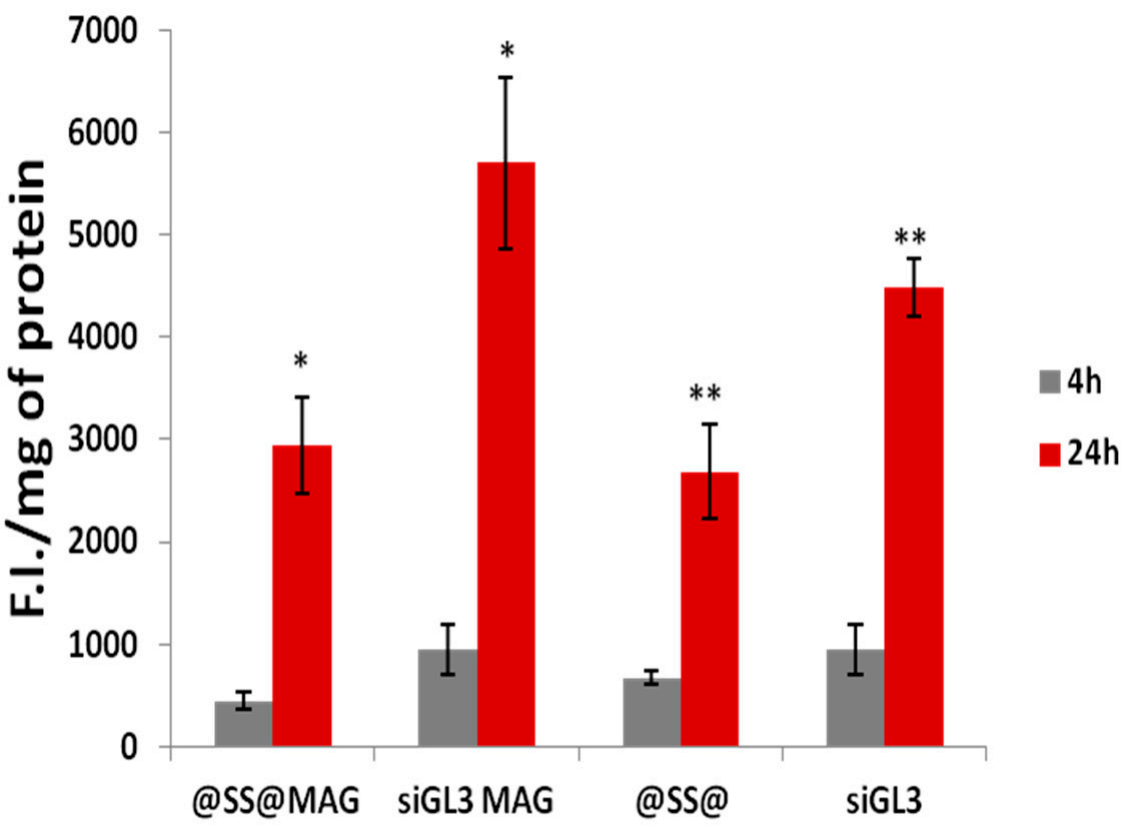
Uptake on MCF-7 cells (4 and 24 h incubation) of ICD@SS@SPIONs/siRNA with (@SS@MAG) and without (@SS@) the application of an external magnetic field. Results were compared with cells treated with free siRNA in the presence (siGL3MAG) or not (siGL3) of the external magnetic field. A statistically significant difference was found between groups headed by the same symbol (*; **) with *p* ≤ 0.05.

Uptake studies were also conducted on the same cell line, under similar conditions (Figure 11).

Surprisingly, we observed that MCF-7 cells internalized siRNA carried by ICD@SS@SPIONs at a comparable extent either in the presence or absence of the applied magnetic field. This finding indicated that extent of cellular uptake was not directly related to silencing activity. Similarly, O’Neill et al. found that despite its higher uptake, cyclodextrin-mediated transfection efficiency was significantly lower than for Lipofectamine 2000 in Caco-2 cells [39]. A potential explanation for this phenomenon is that the systems enter the cells by a different pathway of endocytosis that can be more or less favourable for transfection. In the same time the presence of the permanent magnet used for uptake studies, could also have provoked the inhomogeneous attraction to the bottom of the well of magnetized nanoparticles in solution, thus causing. detachment of part of the cell monolayer.

## 4. Conclusions

In conclusion, in this study we developed INU-C-DETA, a cationic and thiolated copolymer starting from the natural polysaccharide inulin, which was shown to act both as a SPIONs colloidal stabilizer coating, even in high ionic strength medium and in the presence of BSA and as a polycation for siRNA loading. The multistep formulation process here described, involving deprotection of −S−S− groups into pendent thiol by reduction, subsequent SPIONs/INU-C-DETA/siRNA interaction and crosslinking, enabled the formation of magnetically targeted and also redox responsive siRNA delivery systems. These were found to be stable after freeze drying process and to be able to protect siRNA from enzymatic activity. Moreover, they have been shown to be haemo- and cytocompatible and are able to efficiently deliver siRNA into MCF-7 cells together with the ability to increase this effect in the presence of an external magnetic field. Thus, the crosslinked ICD@SS@SPIONs/siRNA formulation could serve as a promising system for addressing several critical challenges in non-viral siRNA delivery.

## Figures and Tables

**Table 1:** Summary of reaction conditions and characterization data of INU-C-DETA.

Polymer	Employed Molar Ratios				Molecular Characteristics			
	*R*_1_	*R*_2_	*R*_3_	*R*_4_	*DD_mol_% C*	*DD_mol_% DETA*	${\bar{M}}_{w}\;$	${\bar{M}}_{w}/{\bar{M}}_{n}\;$
**INU-C-DETA**	0.75	1	3.75	2	5 ± 3	20 ± 2	6217	1.83
**INU**	/	/	/	/	/	/	4782	1.52

**R_1_**: mmol 4-BNPC/mmol fructose repeating units; **R_2_**: mmol C/mmol U.R. Inu; **R_3_**: mmol DETA/mmol fructose repeating units; **R_4_**: mmol TEA/mmol C.

**Table 2:** Composition information.

System	Yield wt%	mmol (SH + S–S)/mg%	Fe_3_O_4_ wt%
ICD@SPIONs	46 ± 7	0.013	8.45
ICD@SS@SPIONs	60 ± 5	0.025	4.40

**Table 3:** Size and ζ potential measured by DLS of ICD@SPIONs (uncrosslinked), ICD@SS@SPIONs (crosslinked) and ICD@SS@SPIONs/siRNA nanoparticles, before (black coloured data) and after (grey coloured data) freeze drying process. PDI values are reported as data labels.

System	Before Freeze Drying		After Freeze Drying			
	H_2_O		H_2_O		PBS	
	Z-Aver/nm (PDI)	ζ-Pot/mV	Z-Aver/nm (PDI)	ζ-Pot/mV	Z-Aver/nm (PDI)	ζ-Pot/mV
ICD@SS@SPIONs	170 (0.093)	9.16	169 (0.203)	8.97	168 (0.151)	–2.7
ICD@SS@SPIONs/siGL3	458 (0.241)	–5.86	467 (0.200)	–5.83	600 (0.243)	–5.6

## Supplementary Materials

The following are available online at https://www.mdpi.com/2073-4360/11/5/889/s1, Figure S1: Photographs of ICD@SS@SPIONs water dispersion during (a) and after (b) 2 h exposition of external magnetic field; Figure S2: Profile of fluorescence in EtBr exclusion assay as a function of system/siRNA weight ratio. Squares: INU-C-DETA/siGL3; Figure S3: Dispersion of nanoparticles in DMEM supplemented with 10v% FBS; Figure S4: Extrapolation of relative fluorescence intensity of BSA saturated with nanoparticles (Fs) for ICD@SS@SPIONs (A) and ICD@SS@SPIONs/siGL3 (B); Figure S5: Microscopic images of RBCs and haemolysis percentage after treatment with ICD@SS@SPIONs (A,B) and INU-C-DETA (C,D); Figure S6a: Cell viability assay on 16HBE cells of INU-C-DETA (A), ICD@SS@SPIONs (B) and ICD@SPIONs (C); Figure S6b: Cell viability assay on MCF-7 cells of INU-C-DETA (A), ICD@SS@SPIONs (B) and ICD@SPIONs (C).
