# Supplementary material for: Combining Inulin Multifunctional Polycation and Magnetic Nanoparticles: Redox-Responsive siRNA-Loaded Systems for Magnetofection
Source: Polymers (Basel). 2019 May 15;11(5):889. doi: 10.3390/polym11050889 (PMC6571810; doi:10.3390/polym11050889)
Supplement: Supplementary file 1 [file polymers-11-00889-s001.pdf]

Supplementary Materials

Combining Inulin Multifunctional Polycation and Magnetic Nanoparticles: Redox-Responsive siRNA-Loaded Systems for Magnetofection

Carla Sardo, Emanuela Fabiola Craparo, Barbara Porsio, Gaetano Giammona and Gennara Cavallaro

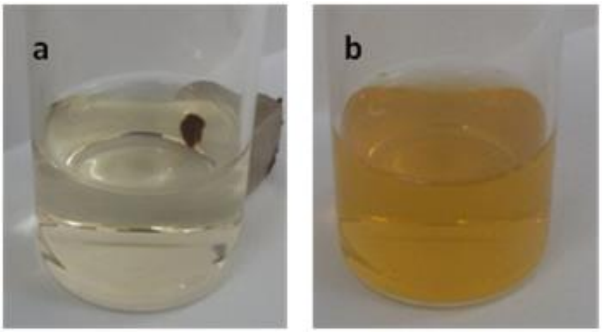

Figure S1. Photographs of ICD@SS@SPIONs water dispersion during (**a**) and (**b**) after 2 h exposition of external magnetic field.

Ethydium Bromide (EtBr) Exclusion Assay: Dose-dependent condensation and encapsulation efficiency of siRNA by INU-C-DETA, ICD@SPIONs and ICD@**SS**@SPIONs was examined by the quenching of EtBr fluorescence in a EtBr exclusion assay. A fixed amount of siRNA (0.5 μg of siRNA in 20 μL of RNAse-free DPBS pH 7.4) was mixed with increasing amounts of copolymer or nanoparticles contained in 20 μL of RNAse-free DPBS pH 7.4, corresponding to 5–40 weight ratios, and incubated at room temperature for 2h. After this time 40 μL EtBr solution 5×10^{-3} mg/mL in DPBS (pH 7.4) were added and the samples were further incubated for 30 min. The fluorescence intensity was measured using a plate reader AF 2200 (Eppendorf, Hamburg, Germany) at an excitation wavelength of 530 ± 25 nm and emission wavelength of 590 ± 10 nm. Results were expressed as a percentage relative to the naked siRNA-EtBr sample.

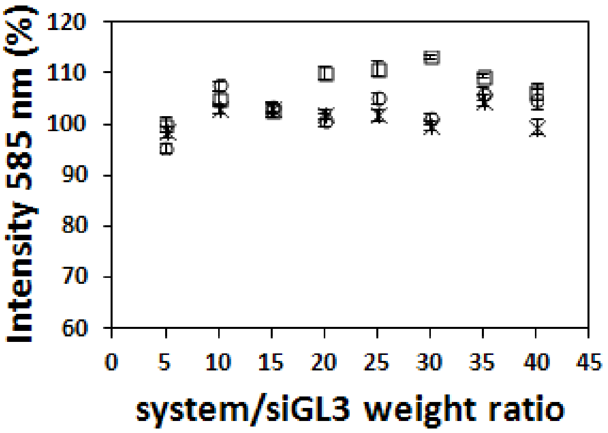

Figure S2. Profile of fluorescence in EtBr exclusion assay as a function of system/siRNA weight ratio. Squares: INU-C-DETA/siGL3; Circles: ICD@SS@SPIONs/siGL3; Asterisks: ICD@SPIONs/siGL3.

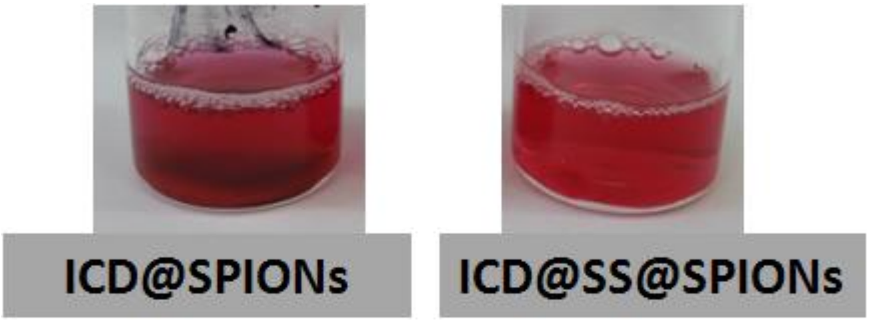

Figure s3. Dispersion of nanoparticles in DMEM supplemented with 10v% FBS.

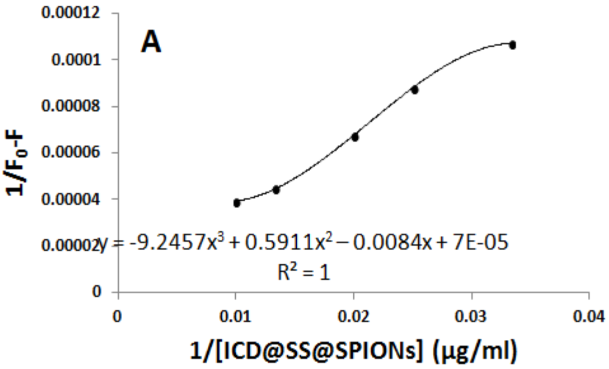

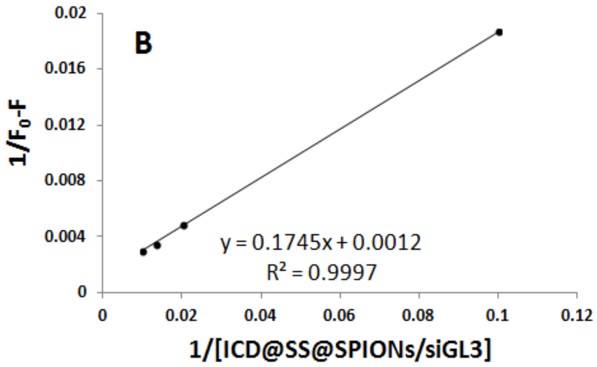

Figure S4. Extrapolation of relative fluorescence intensity of BSA saturated with nanoparticles (Fs) for ICD@SS@SPIONs (**A**) and ICD@SS@SPIONs/siGL3 (**B**).

S5. Materials, Cell Lines Culture and Reagents

Inulin (from dhalia tubers, average polymerization degree = 36, M_w 5000 Da), diethylenetriamine (DETA), cystamine dihydrochloride (C·HCl), triethylamine (TEA), Bis(4-nitrophenyl)carbonate (4-BNPC), Sephadex G-25, anhydrous dimethyl sulfoxide (a-DMSO), dithionitrobenzoic acid (DTNB), picrylsulfonic acid (TNBS), Iron oxide(II,III), magnetic nanoparticles solution 5 mg/mL in H_2O (SPIONs, avg. part. size 9-11 nm measured by TEM; magnetization at room temperature >45 emu/g at 4500 Oe), tris(2-carboxyethyl)phosphine hydrochloride, dithiotretiol (DTT), heparin, iron(III)chloride, ferrozine (3-(2-pyridyl)-5,6-bis(phenyl sulfonic acid)-1,2,4-triazine), neocuproine (2,9-dimethyl(1,10-phenanthroline), L-Ascorbic acid sodium salt, L-Glutathione reduced (GSH) and Bovin Serum Albumin (BSA) were purchased from Sigma Aldrich (Milan, Italy). SpectraPor dialysis tubing was purchased from Spectrum Laboratories (SpectrumLabs now Repligen, Waltham, MA, USA). Pullulan SEC standards (112.0–0.18 kDa) were purchased by Polymer Laboratories (Church Stretton, UK).

Human bronchial epithelial (16HBE) and human breast cancer (MCF-7) cells were purchased from Istituto Zooprofilattico Sperimentale della Lombardia e dell'Emilia Romagna (Italy). Human breast cancer cells stably expressing firefly luciferase gene and neomycin resistant gene (MCF-7/Luc) were purchased from Cell Biolabs, Inc. (San Diego, CA, USA). Cells were grown in Dulbecco's modified Eagle's medium (DMEM) with 10% fetal bovine serum (FBS) and 1% of penicillin/streptomycin (100 U/mL penicillin and 100 mg/mL streptomycin), 1% glutamine and 0.5% of amphoterycin B, at 37 °C in 5% CO_2 humidified atmosphere. DMEM and other constituents were purchased by Euroclone. Opti-MEM® I Reduced-Serum Medium was purchased from Life Technologies (Carlsbad, CA, USA). Cell Titer 96 Aqueous One Solution (MTS reagents for cell proliferation assay) was purchased from Promega Italy. siRNA: antisense and sense with and without Cy5 linked to the 5′ end siRNA strands were purchased from Biomers.net (Ulm, Germany). The strand sequences (5′→3′) are reported herewith below:

Luciferase GL3 sense: CUUACGCUGAGUACUUCGA

Luciferase GL3 antisense: UGCAAGUACUCAGCGUAAG

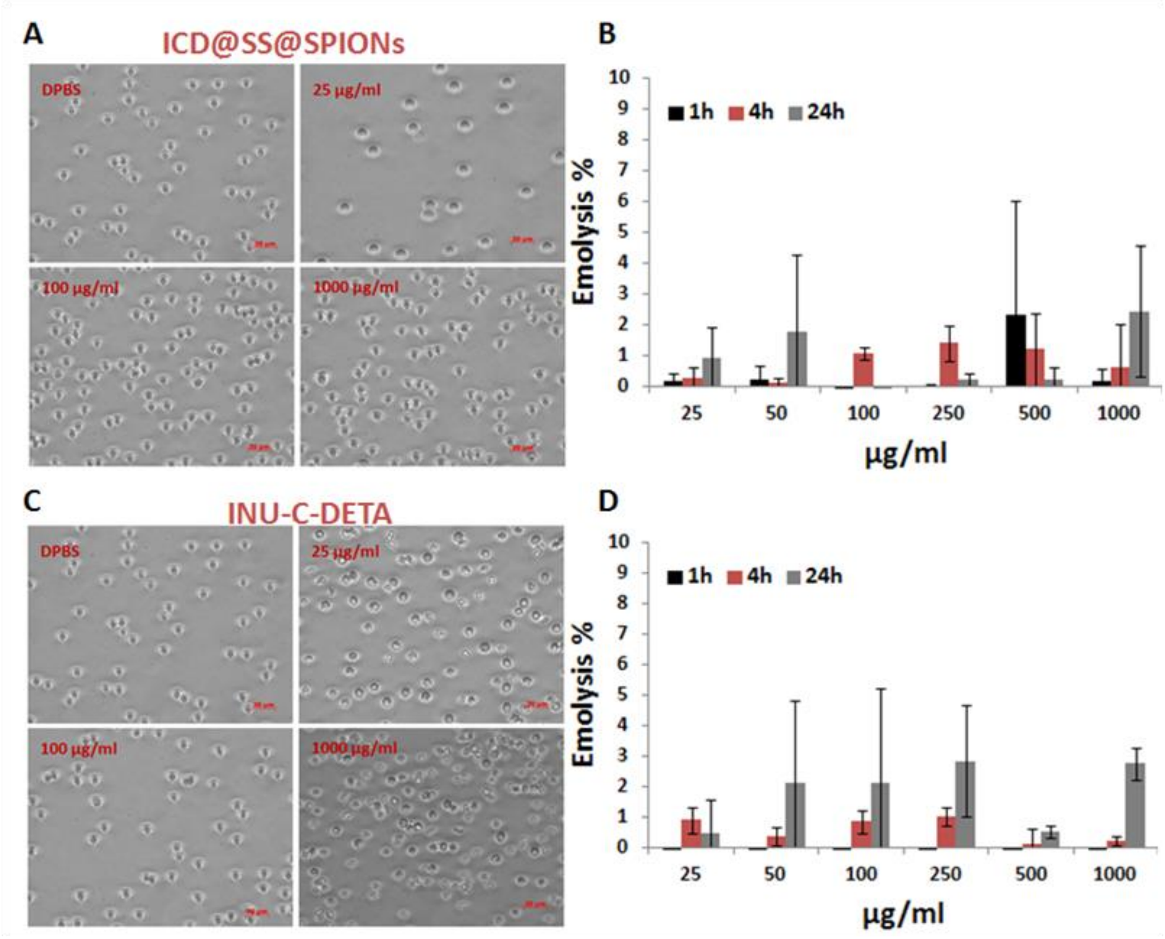

Figure S5. Microscopic images of RBCs and haemolysis percentage after treatment with ICD@**SS**@SPIONs (A-B) and INU-C-DETA (C-D).

Hemocompatibility was assayed by microscopic visualization of human red blood cells (hRBCs) by a Axio Vert.A1 fluorescence microscope (Zeiss, Oberkochen, Germany). The images were recorded using an Axio Cam MRm (Zeiss). Moreover, haemolysis assay according with a previously reported procedure was performed [1]. Complete haemolysis was achieved by using a 1 v% aqueous solution of Triton X-100 (100% control value). Each experiment was performed in triplicate and repeated twice. Results represent mean ± standard deviation for triplicate samples.

S6. Cytotoxicity Assay on 16HBE and MCF-7 Cells

The cytotoxicity was assessed by the MTS cell proliferation assay. Cells were seeded in 96 well plate at a density of 2.5 × 10^4 cells/well and grown as reported above. After 24 h the medium was replaced with 200 µL of fresh DMEM containing INU-C-DETA or ICD@**SS**@SPIONs at various concentrations (25-500 µg/mL). After 48h, DMEM was replaced with 100 µL of fresh medium, and 20 µL of a MTS solution was added to each well. Plates were incubated for additional 2 h at 37 °C. Then, the absorbance at 490 nm was measured using a micro-plate reader (Multiskan, Thermo, UK). Pure cell medium was used as a negative control. Results were expressed as percentage reduction of the control cells. All experiments were performed in triplicates.

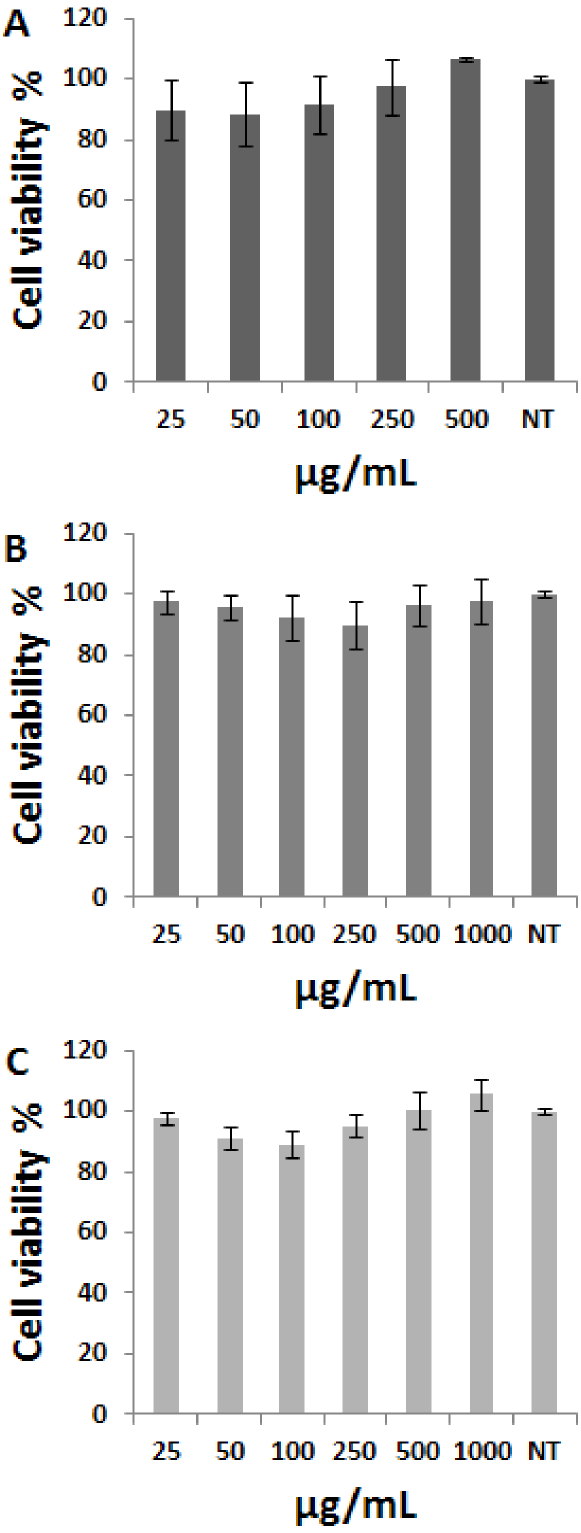

Figure 6a. Cell viability assay on 16HBE cells of INU-C-DETA (**A**), ICD@**SS**@SPIONs (**B**) and ICD@SPIONs (C).

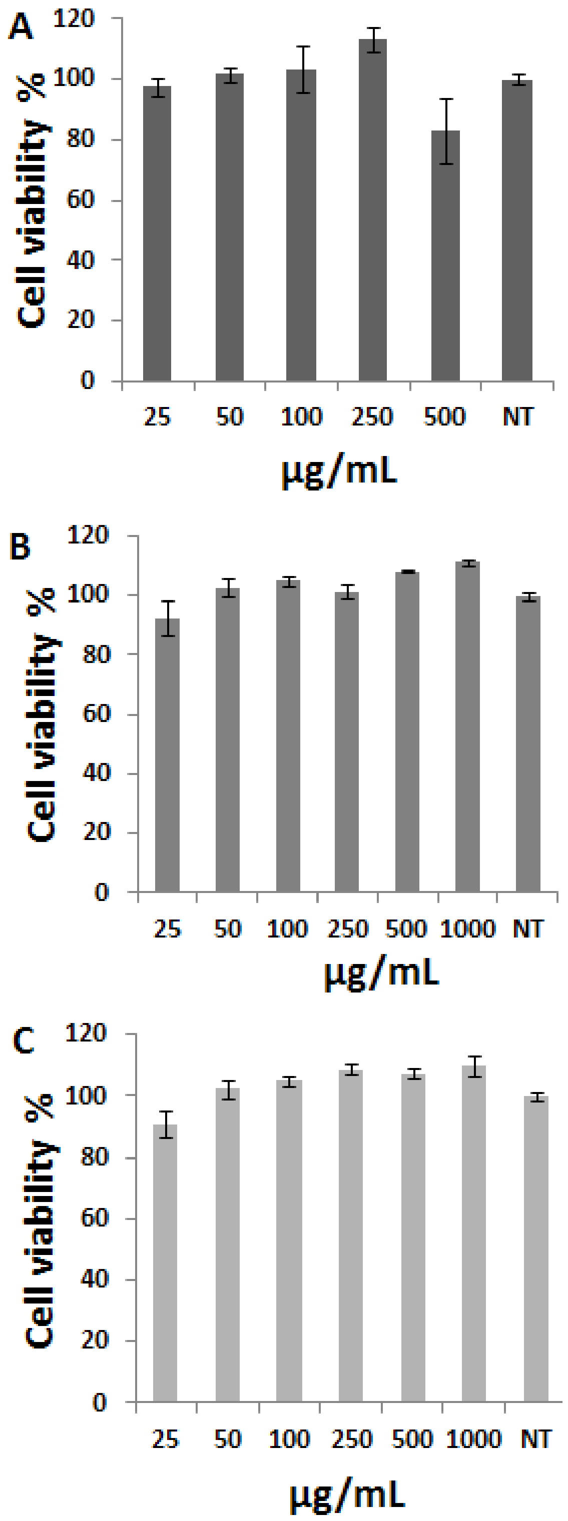

Figure 6b. Cell viability assay on MCF-7 cells of INU-C-DETA (**A**), ICD@**SS**@SPIONs (**B**) and ICD@SPIONs (C).

References

1. Sardo, C.; Craparo, E.F.; Porsio, B.; Giammona, G.; Cavallaro, G. Improvements in Rational Design Strategies of Inulin Derivative Polycation for siRNA Delivery. *Biomacromolecules* **2016**, *17*, 2352, doi:10.1021/acs.biomac.6b00281.
